# Multimodal imaging analyses in patients with genetic and sporadic forms of small vessel disease

**DOI:** 10.1038/s41598-018-36580-0

**Published:** 2019-01-28

**Authors:** Ko Woon Kim, Hunki Kwon, Young-Eun Kim, Cindy W. Yoon, Yeo Jin Kim, Yong Bum Kim, Jong Min Lee, Won Tae Yoon, Hee Jin Kim, Jin San Lee, Young Kyoung Jang, Yeshin Kim, Hyemin Jang, Chang-Seok Ki, Young Chul Youn, Byoung-Soo Shin, Oh Young Bang, Gyeong-Moon Kim, Chin-Sang Chung, Seung Joo Kim, Duk L. Na, Marco Duering, Hanna Cho, Sang Won Seo

**Affiliations:** 1 Department of Neurology, Samsung Medical Center, Sungkyunkwan University School of Medicine, Seoul, Korea; 2 Department of Neurology, Chonbuk National University Medical School & Hospital, Jeonju, Korea; 3 Department of Biomedical Engineering, Hanyang University, Seoul, Korea; 4 Department of Neurology, Yale University School of Medicine, New Haven, Connecticut USA; 5 Genome Research Center, Green Cross Genome, Yong-in, Korea; 6 Department of Neurology, Inha University School of Medicine, Incheon, Korea; 7 Department of Neurology, Chuncheon Sacred Heart Hospital, Hallym University College of Medicine, Chuncheon, Korea; 8 Department of Neurology, Kangbuk Samsung Hospital, Sungkyunkwan University School of Medicine, Seoul, Korea; 9 Department of Neurology, Kyung Hee University Hospital, Seoul, Korea; 10 Department of Neurology, Kangwon National University Hospital, Kangwon National University College of Medicine, Chuncheon, Korea; 11 Department of Laboratory Medicine and Genetics, Samsung Medical Center, Sungkyunkwan University School of Medicine, Seoul, Korea; 12 Department of Neurology, Chung-Ang University College of Medicine, Seoul, Korea; 13 Neuroscience Center, Samsung Medical Center, Seoul, Korea; 14 Department of Clinical Research Design & Evaluation, SAIHST, Sungkyunkwan University, Seoul, Korea; 15 Institute for Stroke and Dementia Research (ISD), University Hospital, LMU, Munich, Germany; 16 Department of Neurology, Gangnam Severance Hospital, Yonsei University College of Medicine, and Departments of, Clinical Research Design and Evaluation, Seoul, Korea

**Keywords:** Cerebrovascular disorders, White matter disease

## Abstract

Cerebral autosomal dominant arteriopathy with subcortical infarcts and leukoencephalopathy (CADASIL) is thought to be a pure genetic form of subcortical vascular cognitive impairment (SVCI). The aim of this study was to compare white matter integrity and cortical thickness between typical CADASIL, a genetic form, and two sporadic forms of SVCI (with *NOTCH3* and without *NOTCH3* variants). We enrolled typical CADASIL patients (N = 11) and SVCI patients [with *NOTCH3* variants (N = 15), without *NOTCH3* variants (N = 101)]. To adjust the age difference, which reflects the known difference in clinical and radiologic courses between typical CADASIL patients and SVCI patients, we constructed a W-score of measurement for diffusion tensor image and cortical thickness. Typical CADASIL patients showed more frequent white matter hyperintensities in the bilateral posterior temporal region compared to SVCI patients (*p* < 0.001, uncorrected). We found that SVCI patients, regardless of the presence of *NOTCH3* variants, showed significantly greater microstructural alterations (W-score, *p* < 0.05, FWE-corrected) and cortical thinning (W-score, *p* < 0.05, FDR-corrected) than typical CADASIL patients. In this study, typical CADASIL and SVCI showed distinct anatomic vulnerabilities in the cortical and subcortical structures. However, there was no difference between SVCI with *NOTCH3* variants and SVCI without *NOTCH3* variants.

## Introduction

Cerebral autosomal dominant arteriopathy with subcortical infarcts and leukoencephalopathy (CADASIL) is characterized by extensive cerebral small vessel disease (CSVD) and cognitive impairments in later life. CADASIL therefore refers to a pure genetic form of subcortical vascular cognitive impairment (SVCI)^1^. CADASIL results from mutations in *NOTCH3*, which is predominantly expressed in vascular smooth muscle cells, preferentially in small arteries. Mutant *NOTCH3* receptors have been proposed to induce the deposition of granular osmiophilic material (GOM) in vascular smooth muscle cells in the small arterioles. This leads to ischemia in the white matter, eventually resulting in CSVD^2^. On the other hand, sporadic SVCI results from vascular risk factors. Specifically, hypertension and diabetes can cause arteriosclerosis in small vessels, which then leads to CSVD, including white matter hyperintensities (WMH) and lacunes on magnetic resonance imaging (MRI)^3^.

Previous studies have shown that genetic forms of dementia have distinct features compared to sporadic forms. Specifically, autosomal dominant Alzheimer’s disease (AD) starts earlier and deteriorates faster than sporadic AD, although autosomal dominant AD and sporadic AD share a common pathophysiological cascade^4,5^. Previous studies have also shown that autosomal dominant AD results in cortical thinning in the parietal region, in contrast to sporadic AD^6^. Autosomal dominant AD also results in increased amyloid uptake, predominantly in the striatum^7–9^. Likewise, CADASIL and SVCI may show distinct distributions of WMH, although CADASIL and SVCI share the same CSVD MRI markers, including WMH and lacunes^10^.

CADASIL displays characteristic abnormalities in the anterior parts of the temporal lobes and the external capsule^11–13^. Anterior pole lesions have high sensitivity and specificity, while external capsule lesions have high sensitivity but low specificity^1^. However, considering that the anterior part of the temporal region is less commonly involved in Asian patients with CADASIL compared to European patients with CADASIL^14,15^, studies in Asian patients of the topographical differences of WMH between CADASIL and SVCI would be worthwhile. Previous neuroimaging studies have shown that patients with typical CADASIL and SVCI exhibit white matter microstructural changes^16–18^ and cortical thinning compared to healthy controls^19–21^. In terms of white matter microstructural changes, a study using tract-based spatial statistics (TBSS) demonstrated that CADASIL patients showed decreased fractional anisotropy (FA) and increased mean diffusivity (MD) values in extensive symmetric areas compared to healthy controls^16^. Also, our previous diffusion tensor imaging (DTI) studies revealed that SVCI patients showed decreased FA values in many brain regions relative to healthy controls^17,18^. In terms of cortical thinning, a previous study showed that the cortical thickness of CADASIL patients was not significantly different from that of the normal control group, but the T2* as measured by 7 Tesla MRI was significantly increased compared to that of the healthy control group^22^. Our studies showed that SVCI patients exhibited cortical thinning in the frontal, perisylvian, basal temporal, and posterior cingulate regions compared with healthy controls^18,20,21^. However, CADASIL and SVCI have not been directly and comprehensively compared.

We previously showed that approximately 13% of all consecutively recruited SVCI patients had *NOTCH3* variants^23^, although they did not meet the clinical criteria for CADASIL. There were no differences in the clinical features between SVCI patients with versus without *NOTCH3* variants, while CADASIL patients did show distinctive clinical features compared to SVCI patients with *NOTCH3* variants. A reasonable hypothesis is thus that CADASIL patients show different WMH distributions, different microstructural change topographies, and different patterns of cortical thinning compared to SVCI patients (with and without *NOTCH3* variants), while SVCI patients with *NOTCH3* variants and patients without *NOTCH3* variants do not show differences in these neuroimaging features. However, little is known regarding the neuroimaging features of CADASIL patients, SVCI with *NOTCH3* variants, and SVCI without *NOTCH3* variants (genetic and sporadic types), which share similar phenotypes. In this regard, we tested and confirmed our new hypothesis that there are differences in neuroimaging features among these three groups.

SVCI patients often showed combined AD pathologies. Our previous study found that 64% to 69% of the SVCI patients had no amyloid plaque pathology, but 31% to 36% of the SVCI patients had amyloid plaque pathology using [11C] Pittsburgh compound B (PiB) positron emission tomography (PET) scanning^24–26^. There were some differences between SVCI with amyloid plaque pathology and SVCI without amyloid amyloid plaque pathology in terms of age, Mini-Mental State Examination (MMSE) score, number of lacunae, and visual ratings of medial temporal lobe atrophy (MTA)^24^. SVCI patients without amyloid plaque pathology were younger and had a greater number of lacunes^26^. The proportion of APOE e4 carriers was higher in SVCI patients with amyloid plaque pathology^26^. Therefore, amyloid plaque pathology should be considered when comparing CADASIL with SVCI.

The aim of this study was to compare white matter integrity and cortical thickness between CADASIL, a genetic form, and two sporadic forms of SVCI (with *NOTCH3* and without *NOTCH3* variants). SVCI patients also underwent [11C] PiB PET scanning that is known to have high specificity and sensitivity for detecting amyloid deposition^27^. In this study, we used the term “typical” CADASIL to distinguish it from SVCI groups with *NOTCH3* variant genes. We hypothesized that typical CADASIL patients would exhibit more severe structural damages in the white matter and grey matter compared to SVCI patients (with *NOTCH3* variants and SVCI patients without *NOTCH3* variants), because genetic forms usually have more severe manifestations than sporadic forms of disease. We also hypothesized that cortical thickness and white matter integrity in SVCI patients with *NOTCH3* variants would not be significantly different from those in SVCI patients without *NOTCH3* variants, considering that a previous study showed that *NOTCH3* variants did not affect clinical features in SVCI patients^23^.

## Results

### Demographic characteristics of participants

The demographic data and clinical features of the patients are described in Table 1. The mean patient age was 57 ± 7 years in the typical CADASIL group, 72 ± 8 years in the SVCI with *NOTCH3* variants group, and 74 ± 7 years in the SVCI without *NOTCH3* variants group. Hypertension was significantly more frequent in the SVCI groups (with and without *NOTCH3* variants) than in the typical CADASIL group. The rates of ischemic stroke occurrence were not significantly different among the typical CADASIL, SVCI with *NOTCH3* variants, and SVCI without *NOTCH3* variants groups. Two patients (13%) in the SVCI with *NOTCH3* variants group were PiB positive, while 32 patients (32%) in the SVCI without *NOTCH3* variants group were PiB positive (Table 1).

**Table 1:** Demographic and clinical features in the typical CADASIL, SVCI with *NOTCH3* variants, and SVCI without *NOTCH3* variants groups.

	Typical CADASIL	SVCI		*p* value
		With *NOTCH3* variants	Without *NOTCH3* variants	
Subjects (number)	11	15	101	
Age, mean ± SD (years)	57 ± 7	72 ± 8	74 ± 7	<0.001
Sex, female, N (%)	5 (45.5)	9 (60)	62 (61.4)	0.592
Education, mean ± SD (years)	12.2 ± 4.6	8.1 ± 5.1	9.0 ± 5.1	0.102
Hypertension (%)	2 (18.2)	13 (86.7)	77 (76.2)	<0.001
Diabetes (%)	3 (27.3)	3 (20.0)	26 (25.7)	0.880
Hyperlipidemia (%)	3 (27.3)	9 (60.0)	30 (29.7)	0.061
Ischemic TIA/stroke (%)	5 (45.5)	4 (26.7)	23 (22.8)	0.256
MMSE, median (IQR)	28 (24–30)	27 (12–30)	25 (9–30)	0.024
PiB-positive (%)	—	2 (13)	32 (32)	0.225

CADASIL: cerebral autosomal dominant arteriopathy with subcortical infarcts and leukoencephalopathy; IQR: interquartile range; MMSE: Mini-Mental State Examination; N: number; PiB: Pittsburgh compound B; SD: standard deviation; SVCI: subcortical vascular cognitive impairment; TIA: transient ischemic attack.

### Spatial distribution of WMH

**Figure 1:**
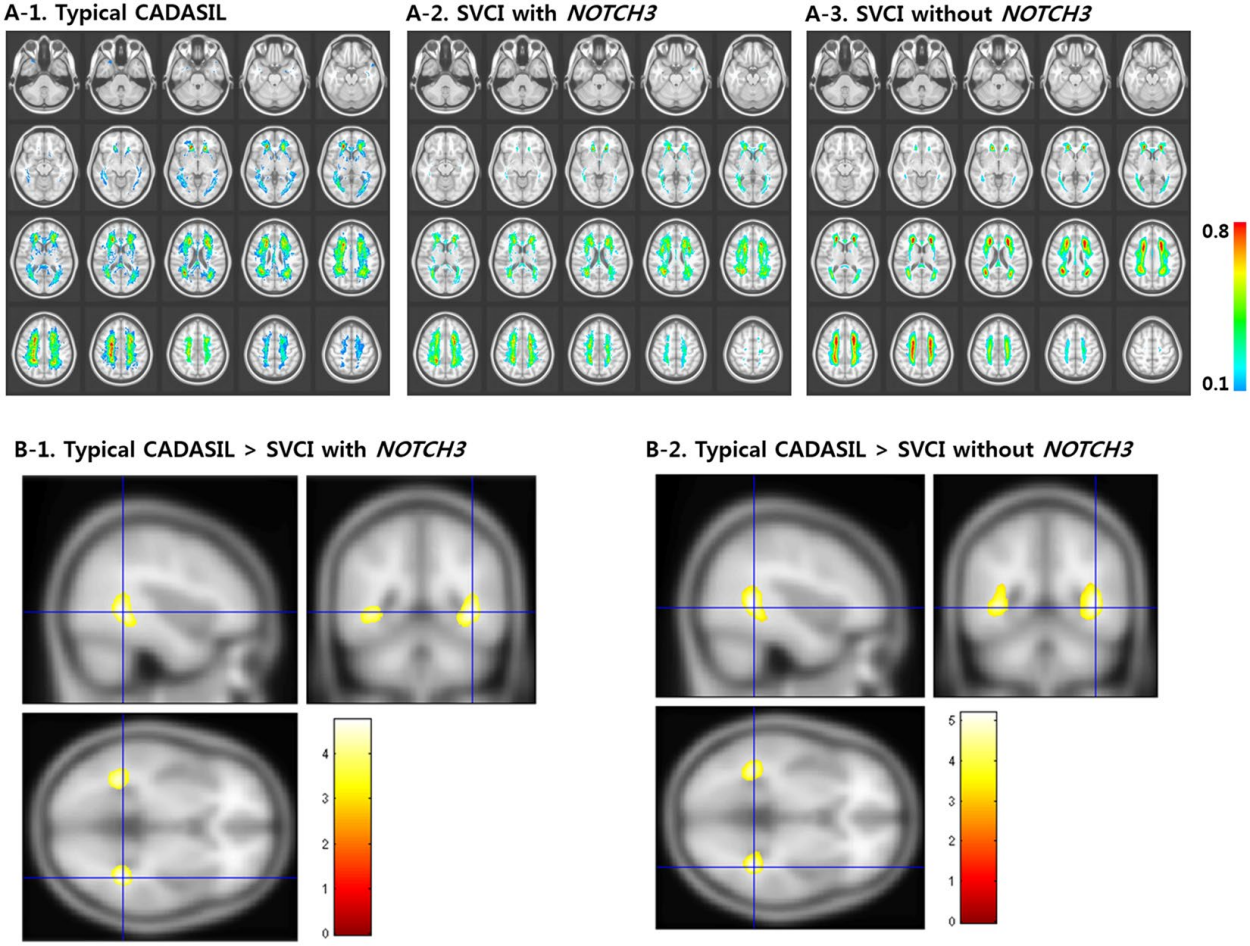
WMH frequency maps of the three groups (**A**) and comparison of frequency maps between the typical CADASIL and SVCI groups (**B**). (**A-1**) Typical CADASIL patients show extensive WMH distributed throughout the periventricle, posterior temporal white matter, and anterior temporal white matter. (**A-2**,**A-3**) SVCI patients with *NOTCH3* variants and SVCI patients without *NOTCH3* variants show similar WMH frequency maps. (**B-1**,**B-2**) Typical CADASIL patients show significantly more prevalent WMH distribution in the bilateral posterior temporal region compared to SVCI patients with *NOTCH3* variants and SVCI patients without *NOTCH3* variants (*p* < 0.001, uncorrected; the corresponding T-value is shown through the color bar.). CADASIL: cerebral autosomal dominant arteriopathy with subcortical infarcts and leukoencephalopathy; SVCI: subcortical vascular cognitive impairment; WMH: white matter hyperintensities.

Figure 1A shows the WMH frequency maps for each of the three groups, which can be used to visualize the spatial distributions of WMH. WMH was most prevalent in the periventricular white matter around the frontal and posterior horns of the lateral ventricles in all three groups (typical CADASIL, SVCI with *NOTCH3* variants, and SVCI without *NOTCH3* variants). As shown in Fig. 1B, WMH was significantly more prevalent in the bilateral posterior temporal region in the typical CADASIL group compared to the two SVCI groups (with and without *NOTCH3* variants) (*p* < 0.001, uncorrected). There was no significant difference between the two SVCI groups (with and without *NOTCH3* variants).

### Microstructural alterations on DTI

**Figure 2:**
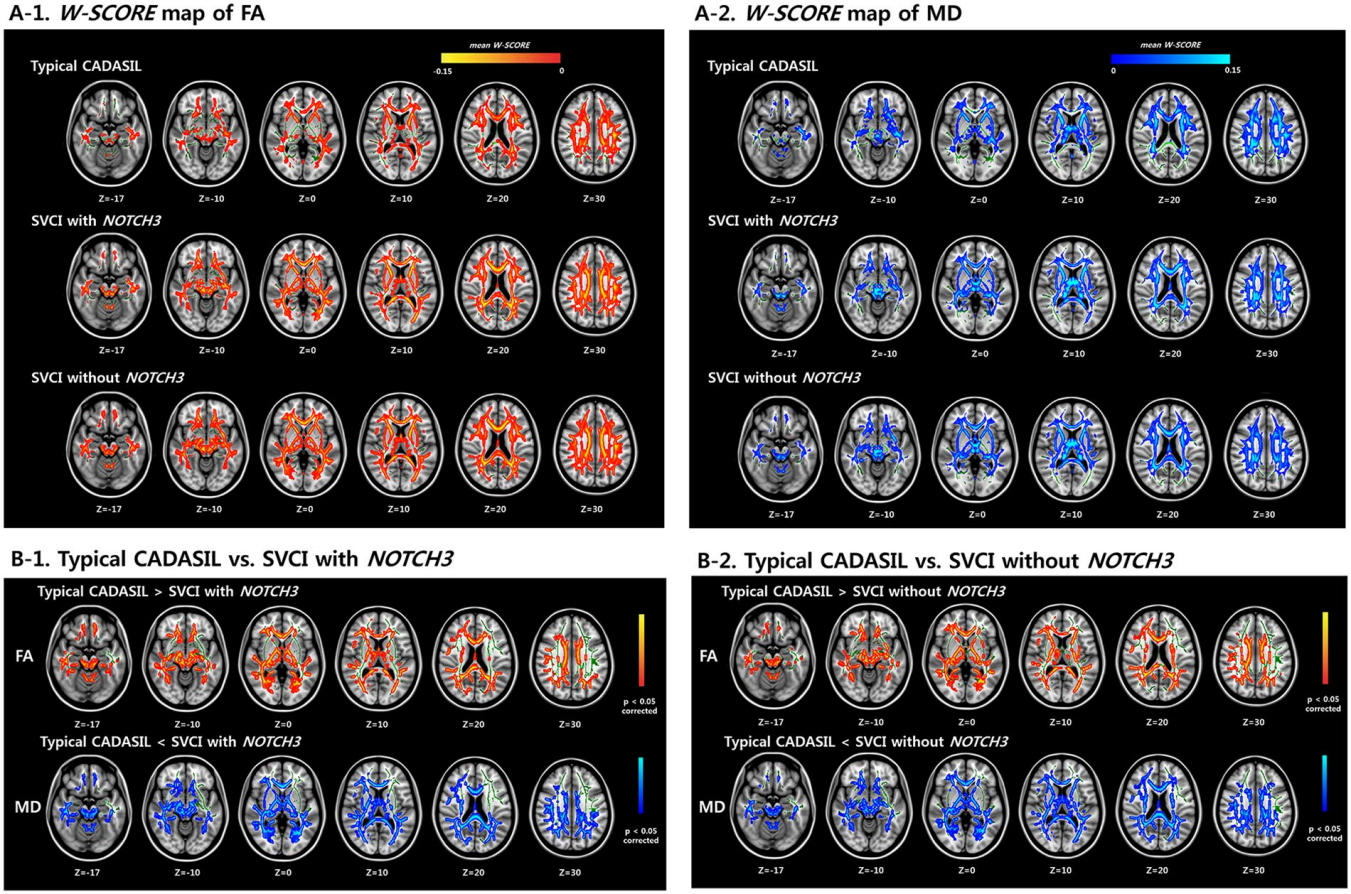
FA and MD W-score maps of the three groups (**A**) and comparison of the W-score maps between the typical CADASIL and SVCI groups (**B**). (**A-1**) Red-yellow colors indicate low W-scores of FA value in typical CADASIL patients, SVCI patients with *NOTCH3* variants, and SVCI patients without *NOTCH3* variants, respectively, in reference to the cognitively normal participants. (**A-2**) Dark to light blue colors indicate higher W-scores of MD value in typical CADASIL patients, SVCI patients with *NOTCH3* variants, and SVCI patients without *NOTCH3* variants, respectively, in reference to the cognitively normal participants. (**B-1**,**B-2**) Red-yellow colors indicate significantly lower W-scores of FA value in SVCI patients (with or without *NOTCH3* variants) compared to the typical CADASIL patients in several brain regions (i.e., white matter in the frontal and parietal lobes, periventricular white matter, temporal white matter, anterior corpus callosum, and posterior corpus callosum). Blue-light blue color indicates significantly higher W-scores of MD value in SVCI patients (with or without *NOTCH3* variants) compared to the typical CADASIL patients in similar regions. These are inversely related to the FA values, suggesting that SVCI patients showed more DTI-measured abnormalities than the typical CADASIL patients (W-score, *p* < 0.05, FWE). However, there are no regions where the typical CADASIL patients showed more DTI-measured abnormalities than the SVCI patients. CADASIL: cerebral autosomal dominant arteriopathy with subcortical infarcts and leukoencephalopathy; FA: fractional anisotropy; MD: mean diffusivity; SVCI: subcortical vascular cognitive impairment.

The W-score maps (Supplementary Fig. 1) of FA and MD showed diffuse microstructural alterations in all three groups [typical CADASIL, SVCI with *NOTCH3* variants, and SVCI without *NOTCH3* variants (Fig. 2A-1,2)]. The W-scores of the FA and MD maps are shown in Fig. 2B-1,2. Both SVCI groups [with *NOTCH3* variants (Fig. 2B-1) and without *NOTCH3* variants (Fig. 2B-2)] showed significantly decreased FA W-scores and significantly fewer MD-involved long white matter tracts in many brain regions, including the bilateral frontal lobe, temporal lobe, parietal lobes, transcallosal fibers, and brainstem, compared to the typical CADASIL group (*p* < 0.05, family-wise error rate [FWE]-corrected). However, there were no regions where the typical CADASIL patients showed more DTI measure abnormalities than the SVCI patients. There was no difference between the two SVCI groups (with and without *NOTCH3* variants).

We next performed a sensitivity analysis for the amyloid-negative group to exclude the possibility that amyloid deposition affected the DTI results. The results confirmed that both SVCI groups (with and without *NOTCH3* variants) showed significantly decreased FA W-scores and significantly fewer MD-involved diffuse white matter tracts compared to the typical CADASIL group (*p* < 0.05, FWE-corrected, Supplementary Fig. 2). Additionally, we analyzed T-value maps for age-matched subjects to remove any potential effect of age on the DTI results. The W-score maps of the FA and MD t-values showed that the SVCI without *NOTCH3* variants group had more diffuse microstructural damage than the typical CADASIL group (Supplementary Fig. 3).

### Topography of cortical thickness

**Figure 3:**
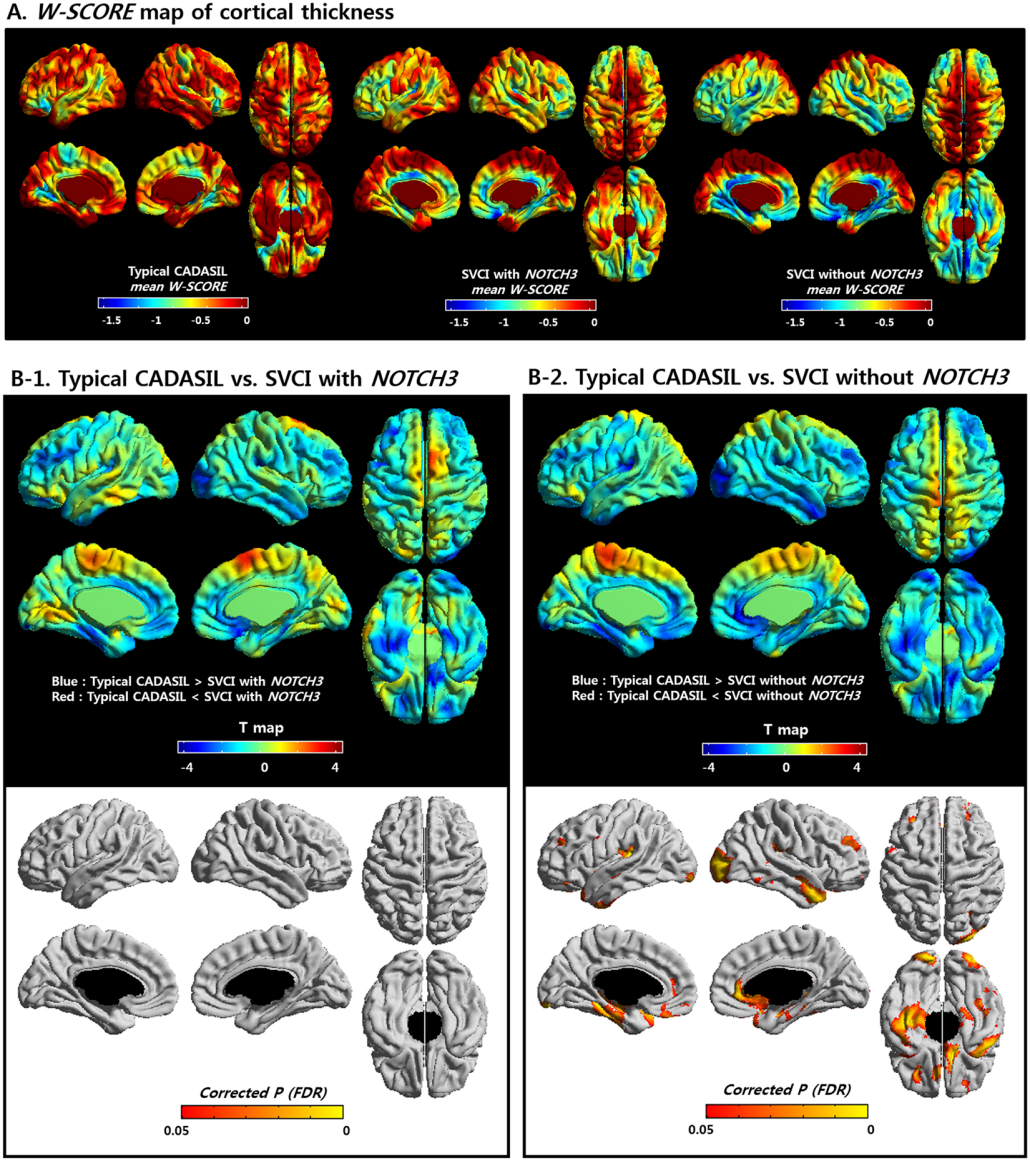
W-scores of cortical thickness patterns of the three groups (**A**) and comparison of the W-scores in the typical CADASIL group and the SVCI groups (**B**). (**A**) Cortical thinning areas in typical CADASIL patients, SVCI patients with *NOTCH3* variants, and SVCI patients without *NOTCH3* variants in reference to cognitively normal participants (W-score). (**B-1**) There is no significant difference in the W-scores of cortical thickness between the SVCI patients with *NOTCH3* variants and the typical CADASIL patients. (**B-2**) SVCI patients without *NOTCH3* variants showed lower W-scores of cortical thickness compared to typical CADASIL patients in the bilateral dorsolateral region, the medial prefrontal region, the occipital region, and the right anterior and left inferior temporal regions, suggesting that SVCI patients without *NOTCH3* variants showed decreased cortical thickness compared to the typical CADASIL patients. CADASIL: cerebral autosomal dominant arteriopathy with subcortical infarcts and leukoencephalopathy; SVCI: subcortical vascular cognitive impairment.

Figure 3A shows the cortical thinning patterns in the three groups as assessed using the W-score. The cortical thickness W-score (Supplementary Fig. 1) for the SVCI without *NOTCH3* variants group was significantly lower than that of the typical CADASIL group in multiple brain regions, including the bilateral dorsolateral region, the medial prefrontal region, the occipital region, and the right anterior and left inferior temporal regions (*p* < 0.05, false discovery rate [FDR]-corrected, Fig. 3B-2). The cortical thickness W-scores for the SVCI with *NOTCH3* variants group showed a similar pattern of decrease compared to those of the typical CADASIL group; however, these differences were not significant (Fig. 3B-1). There were no regions where the typical CADASIL patients showed more decreased cortical thickness than the SVCI patients. There was also no difference between the two SVCI groups (with and without *NOTCH3* variants).

We next performed a sensitivity analysis for the amyloid-negative group to exclude the possibility that amyloid deposition affected cortical thickness. The results confirmed that both SVCI groups (with and without *NOTCH3* variants) showed significantly decreased cortical atrophy compared to the typical CADASIL group; the pattern was the same as that of the main results [*p* < 0.05, false discovery rate (FDR)-corrected, Supplementary Fig. 4]. Another sensitivity analysis was performed with age-matched subjects to ensure that no aging-related effects on cortical thickness were confounding the results; the t-map is shown in Supplementary Fig. 5.

## Discussion

In this study, we investigated spatial features of multimodal neuroimaging among well-characterized cohorts of typical CADASIL patients and SVCI patients with genetic biomarker data. We found that SVCI patients, regardless of the presence of *NOTCH3* variants, showed significantly more microstructural alterations (Fig. 2) and cortical thinning (Fig. 3) than typical CADASIL patients. However, these differences in neuroimaging features were not observed between the SVCI patients with *NOTCH3* variants and those without *NOTCH3* variants.

Our first major finding was that typical CADASIL patients showed more frequent WMH in the bilateral posterior temporal region compared to SVCI patients (Fig. 1). Previously, involvement of WMH in the anterior part of the temporal lobes was suggested to be highly indicative of typical CADASIL^11–13^, with a sensitivity and specificity of 95% and 80%, respectively. However, the reported frequencies of WMH involvement in the anterior temporal region have been lower in studies of Asian participants than in studies of Western participants. Previous studies of Asian participants reported that that 44.8% to 68.4% of all typical CADASIL patients had anterior temporal involvement^15,23,28–30^. Therefore, our findings suggest that Asian CADASIL patients often have WMH involvement in the posterior temporal region (54.5%) as well as in the anterior (54.2%) temporal region.

Our second major finding was that SVCI patients (with *NOTCH3* and without *NOTCH3* variants) had more microstructural alterations in the bilateral frontal lobe, temporal lobe, parietal lobes, anterior corpus callosum, and posterior corpus callosum than typical CADASIL patients (Fig. 2). However, our findings were not in line with a previous study that suggested that CADASIL patients showed more microstructural alterations than patients with small vessel disease^31^. We assumed that our SVCI patients may have more WMH burdens than the patients with small vessel disease included in the previous study because we included SVCI patients with high WMH load only, while the previous study included both low and high WMH load patients. Our suggestion could be supported by subgroup analysis of the previous study, which showed more microstructural alterations in patients with high WMH load compared to ones with low WMH load^31^. Additionally, we performed sensitivity analyses based on amyloid (−) SVCI patients to exclude the possibility that AD pathology might have affected microstructural alterations. Our previous study revealed that SVCI patients often showed combined AD pathologies^24^. Our amyloid (−) SVCI patients also showed results similar to the original analyses (Supplementary Fig. 2). Therefore, unlike a previous study^31^, our findings suggested that sporadic patients with extensive CSVD might have more microstructural alterations than CADASIL patients.

Our third major finding was that the SVCI patients without *NOTCH3* variants showed more cortical thinning in the bilateral dorsolateral region, the medial prefrontal region, the occipital region, and the right anterior and left inferior temporal regions compared to typical CADASIL patients (Fig. 3B-2). In Fig. 3B-1, the T-map shows similar patterns of differences between the typical CADASIL and the SVCI patients with *NOTCH3* variants. However, there was no significant difference between the two groups. This might be related to the small sample size of SVCI patients with *NOTCH3* variants (N = 15). Previous studies have compared the cortical thickness of normal controls with that of CADASIL or SVCI patients. A previous study showed that, while the cortical thickness of typical CADASIL patients was not significantly different from that of the normal control group, the T2* as measured by 7 Tesla MRI was significantly increased compared to that of the normal control group^22^. In our previous study, SVCI patients exhibited cortical thinning in the frontal, perisylvian, basal temporal, and posterior cingulate regions compared with normal controls^18,20,21^. However, the cortical thickness of typical CADASIL and SVCI patients has not been directly and comprehensively compared. In this study, direct comparisons revealed that patients with SVCI without *NOTCH3* variants had more cortical thinning compared to patients with typical CADASIL. Furthermore, we performed sensitivity analyses based on amyloid (−) SVCI patients to exclude the possibility that AD pathology might have affected cortical thickness. Our amyloid (−) SVCI patients also showed that the SVCI patients without *NOTCH3* variants showed more cortical thinning than typical CADASIL patients in patterns similar to the original analyses (Supplementary Fig. 4).

The reason SVCI patients have more abnormalities in their microstructural changes and cortical thinning than typical CADASIL patients remains unknown. Considering that genetic models such as familial Alzheimer’s disease generally have similar but more severe deterioration, both clinically and pathologically^4,5,32^, our findings are unexpected. A possible explanation is that the SVCI patients were, on average, older than the typical CADASIL patients and had more coexistent AD pathologies. However, our sensitivity analyses excluded these potential explanations. Our SVCI patients had significant vascular burden and cognitive impairments, which may limit the generalizability of our data to other populations. Further studies are needed to explain the different pathogenesis mechanisms driving typical CADASIL and SVCI.

Another noteworthy finding with respect to microstructural changes and cortical thickness was that *NOTCH3* variations did not affect WMH distribution or topography in the SVCI patients. Consistent with our finding, a previous study that assessed *NOTCH3* variants revealed that only one of 39 variants was associated with an increased risk of ischemic stroke with small vessel disease subtype in Caucasians^33^. Another study found no association of any common variants in *NOTCH3* with lacunar stroke or WMH volume in Caucasian patients with ischemic stroke^34^. In contrast, a study based on an Austrian population without stroke showed that *NOTCH3* variants were associated with the presence and progression of WMH^35^. Another study showed that only some of the *NOTCH3* variants were associated with ischemic stroke in Chinese patients^36^. Therefore, most *NOTCH3* variants do not seem to be associated with ischemic lesions, although there is a difference depending on race or type of polymorphism. Based on molecular genetics, CADASIL is caused by mutations in *NOTCH3* that lead to an odd number of cysteine residues of epidermal growth factor repeats^11^. However, *NOTCH3* variants did not affect WMH distribution or topography in our study because they do not involve a cysteine residue.

One strength of our study is that we performed multimodal neuroimaging analyses to investigate spatial features among well-characterized cohorts of typical CADASIL patients and SVCI patients with genetic biomarker data. However, our study also had several limitations. First, an inevitable age gap was present between the typical CADASIL and the SVCI groups. Although we analyzed W-scores after controlling for age, the age difference could have affected the microstructural changes and cortical thinning. This argument is mitigated to some degree by the sensitivity analysis comparison, which showed that the two groups with similar ages had similar results. Second, the sample sizes of the typical CADASIL group and the SVCI with *NOTCH3* variants group were small. Despite this limitation, we identified different neuroimaging features of typical CADASIL patients and SVCI patients by directly comparing these two groups.

In conclusion, SVCI patients and typical CADASIL patients showed distinct anatomic vulnerabilities of the grey matter and white matter structures. In contrast, no significant differences were observed between SVCI patients with *NOTCH3* variants and SVCI patients without *NOTCH3* variants. Therefore, our findings suggest that SVCI patients have different pathophysiology compared to typical CADSIL patients. Postmortem studies could help shed light on the reasons for these different involvement patterns.

## Methods

### Subjects

We recruited 11 patients with typical CADASIL and 116 patients with SVCI. The patients with typical CADASIL met the criteria for probable CADASIL, with some modifications^37^. The inclusion criteria were: extensive leukoaraiosis on brain MRI and at least one of the following: young age (<50) at onset of stroke or cognitive decline or a family history of stroke or dementia suggesting autosomal dominant inheritance. In all cases, diagnosis was confirmed by identification of a typical mutation in the *NOTCH3* gene. To be diagnosed with SVCI, patients had to meet the following criteria: (1) a subjective cognitive complaint by the patient or caregiver; (2) an objective cognitive impairment below the 16^th^ percentile in any domain on neuropsychological tests; (3) significant ischemia on brain MRI, defined as periventricular WMH ≥ 10 mm and deep WMH ≥ 25 mm as modified from the Fazekas ischemia criteria^24,38^; and (4) focal neurologic symptoms or signs. In our previous study, 16 of the 117 (13.7%) patients with SVCI had *NOTCH3* variants^23^, although these patients did not meet the probable CADASIL criteria proposed by Davous *et al*.^37^. A known pathogenic mutation was identified in 10 patients with SVCI, and variants of unknown significance (VUS) were identified in 6 patients with SVCI.

All SVCI patients completed a standardized [11C] PiB-PET scan at Samsung Medical Center using a Discovery STE PET/CT scanner (GE Medical Systems, Milwaukee, WI, USA). The detailed radiochemistry profiles, scanning protocol, and data analysis methods are described below. The specific radioactivity of 11C-PiB at the time of administration was more than 1,500 Ci/mmol for patients, and the radiochemical yield was more than 35%. The radiochemical purity of the tracer was more than >95% in all PET studies. The 11C-PiB was injected into an antecubital vein as a bolus with a mean dose of 420 MBq (i.e., range 259–550 MBq). A CT scan was performed for attenuation correction at 60 minutes after the injection. A 30-minute emission static PET scan was then initiated. We calculated the PiB uptake ratio of each voxel using the cerebellum as a reference region in the analysis. The global cortical PiB uptake ratio was determined by combining the bilateral frontal, parietal, and temporal cortices and the posterior cingulate gyrus except the primary motor or sensory cortex. We did not perform partial volume correction. Patients were considered PiB-positive if their global PiB uptake ratio was more than >1.5 (Supplementary Fig. 6)^24^.

We also recruited 56 cognitively normal participants who had no history of neurologic or psychiatric illnesses and no abnormalities detected during neurological examination. They were classified to be cognitively normal by neuropsychological testing. All cognitively normal participants had no or mild WMH (periventricular WMH <10 mm and deep WMH <10 mm in maximal diameter) on MRI.

We excluded patients with territorial infarctions and those with high signal abnormalities on MRI due to radiation injury, multiple sclerosis, vasculitis, or leukodystrophy. All patients completed a clinical interview and neurological examination, as described previously^24^. Additionally, one typical CADASIL patient, two SVCI patients with *NOTCH3* variants, and three SVCI patients without *NOTCH3* variants were excluded due to MRI preprocessing errors. Therefore, the final set of participants analyzed consisted of 10 typical CADASIL patients, 13 SVCI patients with *NOTCH3* variants, and 98 SVCI patients without *NOTCH3* variants.

### Standard protocol approvals, registrations, and patient consents

We obtained written informed consent from each participant. This study was approved by the Institutional Review Board at Samsung Medical Center. In addition, all methods were carried out in accordance with the approved guidelines.

### Molecular genetic analysis

Peripheral blood specimens were collected after informed consent was obtained. Genomic DNA (gDNA) was extracted using a Wizard Genomic DNA Purification Kit according to the manufacturer’s instructions (Promega, Madison, WI, USA). Mutational hotspots of the *NOTCH3* gene, including exons 2–6, 8, 11, 18, 19, and 22, were sequenced. Cycle sequencing was performed with a BigDye Terminator Cycle Sequencing Ready Reaction Kit (Applied Biosystems, Foster City, CA, USA) on an ABI 3130xl Genetic Analyzer (Applied Biosystems). As described in our previous study, we amplified the tested exons in the *NOTCH3* gene and their exon-intron boundaries by polymerase chain reaction, after which we numbered the *NOTCH3* cDNA nucleotides according to a reference sequence^39^, GenBank accession number NM_000435.2. The Sorting Intolerant From Tolerant (SIFT)^40^ and the Polymorphism Phenotyping (PolyPhen-2 v2.1)^41^ servers were used to predict the effects of nonsynonymous VUS on protein structure, function, phenotype, and/or sequence conservation.

### MRI data acquisition

MRI was performed using a 3.0-Tesla MRI scanner (Achieva; Philips Medical Systems, Best, The Netherlands) at Samsung Medical Center. Standardized T2, 3D T1 turbo field echo, 3D fluid-attenuated inversion recovery (fluid-attenuated inversion recovery [FLAIR]), and DTI images were acquired from all applicable participants. Three-dimensional (3D) T1 Turbo Field Echo was acquired using the following imaging parameters: sagittal slice thickness, 1.0 mm, over contiguous slices with 50% overlap; no gap; repetition time^34^, 9.9 ms; echo time (TE), 4.6 ms; flip angle, 8°; and matrix size, 240 × 240 pixels, reconstructed to 480 × 480 over a field of view (FOV) of 240 mm. The following parameters were used for the 3D FLAIR images: axial slice thickness of 2 mm; no gap; TR of 11,000 msec; TE of 125 msec; flip angle of 90°; and matrix size of 512 × 512 pixels. In whole-brain DT-MRI examination, sets of axial diffusion-weighted single-shot echo-planar images were collected with the following parameters: 128 × 128 acquisition matrix, 1.72 × 1.72 × 2 mm^3^ voxel size; 70 axial slices; 22 × 22 cm^2^ field of view; TE 60 ms, TR 7,696 ms; flip angle 90°; slice gap 0 mm; b-factor of 600 smm^−2^. Diffusion-weighted images were acquired in 45 different directions using the baseline image without weighting [0, 0, 0]. All axial sections were acquired parallel to the anterior commissure-posterior commissure. Reproducibility scans were not performed in this study.

### WMH data analysis

A mask of regional WMH was quantified using a pre-developed automated pipeline as previously described^42^. Briefly, candidate WMH (i.e., region with high intensity compared to normal white matter regions) was extracted in the FLAIR image using the FMRIB Automatic Segmentation Tool. To eliminate misclassified regions, the candidate WMH was transformed to the T1-weighted MRI image using the transformation matrix of the affine registration from FLAIR to T1-weighted MRI. Next, the misclassified regions in the candidate WMH were removed by considering the white matter mask acquired from T1-weighted MRI. Thus, the final WMH result was extracted from the T1-weighted MRI space.

### Tract-based spatial statistics (TBSS) analysis of DTI data

DTI is a neuroimaging technique that makes it possible to map microstructural changes of white matter in the brain^43^. FA and MD are common DTI measures. FA is a measure of white matter integrity, and MD is sensitive to cellularity, edema, and necrosis. DTI data were processed using software in the FMRIB Software Library (http://www.fmrib.ox.ac.uk/fsl). Motion artifacts and eddy current distortions were corrected by normalizing each diffusion-weighted volume to the non-diffusion-weighted volume (b0) using the affine registration method in the FMRIB’s Linear Image Registration Tool (FLIRT). Diffusion tensor matrices from the sets of diffusion-weighted images were generated using a general linear fitting algorithm. Subsequently, FA and MD were calculated for every voxel according to standard methods. The FA and MD maps of the DTI preprocessing results were used in TBSS analysis^44^. All FA images were aligned onto a standard FMRIB58 FA template provided by the FSL software, using a nonlinear registration algorithm implemented in the TBSS package. The FA images aligned on the FMRIB 58 FA template were averaged to create a skeletonized mean FA image. Each subject’s aligned FA image was projected onto the skeleton, filling it with the highest FA values from the nearest relevant center of the fiber tracts. A threshold FA value of 0.2 was chosen to exclude voxels of adjacent gray matter or cerebrospinal fluid. MD images were also processed by applying the FA non-linear registration and projecting them onto the skeleton using identical projection methods to those inferred from the original FA data.

### Cortical thickness analysis

The CIVET anatomical pipeline was used to extract cortical thickness (http://mcin-cnim.ca/neuroimagingtechnologies/civet/)^45^. In brief, surfaces of the inner and outer cortices were generated for each hemisphere after correction for intensity non-uniformity, normalization to the MNI 152 template, removal of non-brain tissues, and tissue classification of white matter, gray matter, cerebrospinal fluid, and background^46–50^. The presence of extensive WMH in the MRI scans made it difficult to completely delineate the inner cortical surface with the correct topology due to tissue classification errors. To overcome this technical limitation, we automatically defined the WMH region using a FLAIR image and substituted it for the intensity of peripheral, normal-appearing tissue on the high-resolution T1 image after affine co-registration, as described in earlier studies^42^. Cortical thickness was measured as the Euclidean distance between linked vertices of the inner and outer surfaces that contained 40,962 vertices on each hemisphere in native space^51^. To compare thickness across subjects, the thicknesses were spatially registered to a group template^52,53^ and smoothed with a full-width half-maximum of 20 mm^51^. Intracranial volume (ICV) was calculated by measuring the total volume of gray matter, white matter, and cerebrospinal fluid. Further image processing steps for cortical thickness have been described in previous studies^54–57^.

### Construction of the W-score map

A limitation of this study was the marked age difference in the patient cohorts, which reflects the known difference in clinical and radiologic courses between typical CADASIL patients and SVCI patients. Therefore, we constructed W-score maps to quantitate the degree of WM microstructural changes and cortical atrophy in each patient using DTI measures (i.e., FA, MD) and cortical thickness, based on the cognitively normal participants group as a reference. The W-score concept and computation thereof are described in detail in a previous study^58^. In this study, W-score maps were computed vertex-wise on the surface model and voxel-wise on the skeletonized volume of each imaging data set as follows:

$${\rm{W}}-{\rm{score}}\,=\,\frac{[(patient^{\prime} s\,raw\,value)-(value\,expected\,in\,the\,control\,group\,for\,the\,patient^{\prime} s\,age,sex,\,and\,ICV)]}{SD\,of\,the\,residuals\,in\,controls}$$

W-scores are similar to Z-scores, which have a mean value of 0 and an SD of 1 in cognitively normal participants; values of +1.65 and −1.65 correspond to the 95^th^ and 5^th^ percentiles, respectively. However, W-scores are adjusted for specific covariates such as age, sex, and ICV. To avoid confusion with respect to W-score direction in cortical thickness and DTI measures, we used the W-scores as positive values indicating larger cortical thickness, larger values of FA, and large values of MD^59^.

### Statistics

We examined the normality of the W-scores using a Kolmogorov-Smirnov test. We found that the W-score of mean cortical thickness, mean FA values, and mean MD values satisfied normality (*p* = 0.076~0.200). The difference in variability of W-score between groups was tested with Bartlett’s test for equality of variances. There was no difference in variability of mean cortical thickness values (*p* = 0.0756), mean FA values (*p* = 0.060), and mean MD values (*p* = 0.086). A value of *p* < 0.05 (two-sided) was considered statistically significant.

WMH, global mean DTI W-scores, and cortical thickness data were compared between patient groups by ANCOVA, after the data were adjusted for age and sex, with the Bonferroni post hoc test. To examine the local degrees of atrophy, individual W-score maps were averaged in each patient group. Differences in W-scores at each vertex and each skeletonized voxel following contrast were compared between the following groups: (1) SVCI without *NOTCH3* variants vs. SVCI with *NOTCH3* variants; (2) typical CADASIL vs. SVCI without *NOTCH3* variants; and (3) typical CADASIL vs. SVCI with *NOTCH3* variants. Multiple comparisons were corrected by FDR for cortical thickness or by FWE for microstructural changes (*p* < 0.05). FWE and FDR are methods of conducting multiple comparison correction. Statistical analyses were implemented using the SurfStat toolbox (http://www.math.mcgill.ca/keith/surfstat/), MATLAB (R2012a, The MathWorks, Inc., Natick, MA) and the Randomise function (part of FSL). The resulting t-value and *p*-value maps were projected on an ICBM 152 surface template and a volume template for visualization. Additionally, subgroup analysis was performed for the amyloid-negative group to rule out potential effects of amyloid deposition on cortical thickness.

## Electronic supplementary material

Supplementary Figures

## Data Availability

The datasets generated and/or analyzed during the current study are available from the corresponding author on reasonable request.

## References

[1] Di Donato I (2017). Cerebral Autosomal Dominant Arteriopathy with Subcortical Infarcts and Leukoencephalopathy (CADASIL) as a model of small vessel disease: update on clinical, diagnostic, and management aspects. BMC Med.

[2] Chabriat H (1999). Clinical severity in CADASIL related to ultrastructural damage in white matter: *in vivo* study with diffusion tensor MRI. Stroke.

[3] Roman GC, Erkinjuntti T, Wallin A, Pantoni L, Chui HC (2002). Subcortical ischaemic vascular dementia. Lancet Neurol.

[4] Bateman RJ (2012). Clinical and Biomarker Changes in Dominantly Inherited Alzheimer’s Disease. New Engl J Med.

[5] Jack CR (2010). Hypothetical model of dynamic biomarkers of the Alzheimer’s pathological cascade. Lancet Neurol.

[6] Weston PS (2016). Presymptomatic cortical thinning in familial Alzheimer disease: A longitudinal MRI study. Neurology.

[7] Klunk WE (2007). Amyloid deposition begins in the striatum of presenilin-1 mutation carriers from two unrelated pedigrees. J Neurosci.

[8] Koivunen J (2008). PET amyloid ligand [11C]PIB uptake shows predominantly striatal increase in variant Alzheimer’s disease. Brain.

[9] Remes AM (2008). Carbon 11-labeled pittsburgh compound B positron emission tomographic amyloid imaging in patients with APP locus duplication. Arch Neurol.

[10] O’Brien JT, Thomas A (2015). Vascular dementia. Lancet.

[11] Chabriat H, Joutel A, Dichgans M, Tournier-Lasserve E, Bousser MG (2009). Cadasil. Lancet Neurol.

[12] Auer DP (2001). Differential lesion patterns in CADASIL and sporadic subcortical arteriosclerotic encephalopathy: MR imaging study with statistical parametric group comparison. Radiology.

[13] O’Sullivan M (2001). MRI hyperintensities of the temporal lobe and external capsule in patients with CADASIL. Neurology.

[14] Lee JS (2011). Effects of Lacunar Infarctions on Cognitive Impairment in Patients with Cerebral Autosomal-Dominant Arteriopathy with Subcortical Infarcts and Leukoencephalopathy. J Clin Neurol.

[15] Kim Y (2006). Characteristics of CADASIL in Korea: a novel cysteine-sparing Notch3 mutation. Neurology.

[16] Mascalchi M (2017). Diffusion Tensor Imaging to Map Brain Microstructural Changes in CADASIL. J Neuroimaging.

[17] Jung NY (2016). Tract-Specific Correlates of Neuropsychological Deficits in Patients with Subcortical Vascular Cognitive Impairment. J Alzheimers Dis.

[18] Kim YJ (2015). White matter microstructural changes in pure Alzheimer’s disease and subcortical vascular dementia. Eur J Neurol.

[19] Jouvent E (2012). Longitudinal changes of cortical morphology in CADASIL. Neurobiol Aging.

[20] Kim CH (2012). Cortical thinning in subcortical vascular dementia with negative 11C-PiB PET. J Alzheimers Dis.

[21] Kim HJ (2014). Cortical thickness and hippocampal shape in pure vascular mild cognitive impairment and dementia of subcortical type. Eur J Neurol.

[22] De Guio F (2014). *In vivo* high-resolution 7 Tesla MRI shows early and diffuse cortical alterations in CADASIL. PLoS One.

[23] Yoon CW (2015). NOTCH3 variants in patients with subcortical vascular cognitive impairment: a comparison with typical CADASIL patients. Neurobiol Aging.

[24] Lee JH (2011). Identification of pure subcortical vascular dementia using 11C-Pittsburgh compound B. Neurology.

[25] Lee MJ (2014). Synergistic effects of ischemia and beta-amyloid burden on cognitive decline in patients with subcortical vascular mild cognitive impairment. JAMA psychiatry.

[26] Kim GH (2014). Seoul criteria for PiB(−) subcortical vascular dementia based on clinical and MRI variables. Neurology.

[27] Klunk WE (2004). Imaging brain amyloid in Alzheimer’s disease with Pittsburgh Compound-B. Ann Neurol.

[28] Liao YC (2015). Characterization of CADASIL among the Han Chinese in Taiwan: Distinct Genotypic and Phenotypic Profiles. PLoS One.

[29] Liu X (2015). The genetic spectrum and the evaluation of CADASIL screening scale in Chinese patients with NOTCH3 mutations. J Neurol Sci.

[30] Yin X (2015). Cerebral autosomal dominant arteriopathy with subcortical infarcts and leukoencephalopathy: Phenotypic and mutational spectrum in patients from mainland China. Int J Neurosci.

[31] Baykara E (2016). A Novel Imaging Marker for Small Vessel Disease Based on Skeletonization of White Matter Tracts and Diffusion Histograms. Ann Neurol.

[32] Shinohara M (2014). Regional distribution of synaptic markers and APP correlate with distinct clinicopathological features in sporadic and familial Alzheimer’s disease. Brain.

[33] Ross OA (2013). NOTCH3 variants and risk of ischemic stroke. PLoS One.

[34] Rutten-Jacobs LCA (2015). Common NOTCH3 Variants and Cerebral Small-Vessel Disease. Stroke.

[35] Schmidt H (2011). Genetic variants of the NOTCH3 gene in the elderly and magnetic resonance imaging correlates of age-related cerebral small vessel disease. Brain.

[36] Yuan X, Dong Z (2016). The Association Between the Genetic Variants of the NOTCH3 Gene and Ischemic Stroke Risk. Med Sci Monit.

[37] Davous P (1998). CADASIL: a review with proposed diagnostic criteria. Eur J Neurol.

[38] Fazekas F, Chawluk JB, Alavi A, Hurtig HI, Zimmerman RA (1987). MR signal abnormalities at 1.5 T in Alzheimer’s dementia and normal aging. AJR Am J Roentgenol.

[39] Kim YE (2014). Spectrum of NOTCH3 mutations in Korean patients with clinically suspicious cerebral autosomal dominant arteriopathy with subcortical infarcts and leukoencephalopathy. Neurobiol Aging.

[40] Ng PC, Henikoff S (2001). Predicting deleterious amino acid substitutions. Genome research.

[41] Ramensky V, Bork P, Sunyaev S (2002). Human non-synonymous SNPs: server and survey. Nucleic acids research.

[42] Jeon S (2011). Fully Automated Pipeline for Quantification and Localization of White Matter Hyperintensity in Brain Magnetic Resonance Image. Int J Imag Syst Tech.

[43] Alexander AL, Lee JE, Lazar M, Field AS (2007). Diffusion tensor imaging of the brain. Neurotherapeutics.

[44] Smith SM (2006). Tract-based spatial statistics: voxelwise analysis of multi-subject diffusion data. Neuroimage.

[45] Zijdenbos AP, Forghani R, Evans AC (2002). Automatic “pipeline” analysis of 3-D MRI data for clinical trials: application to multiple sclerosis. IEEE transactions on medical imaging.

[46] Collins DL, Neelin P, Peters TM, Evans AC (1994). Automatic 3D intersubject registration of MR volumetric data in standardized Talairach space. Journal of computer assisted tomography.

[47] Zijdenbos A (1996). Automatic quantification of multiple sclerosis lesion volume using stereotaxic space. Lect Notes Comput Sc.

[48] Sled JG, Zijdenbos AP, Evans AC (1998). A nonparametric method for automatic correction of intensity nonuniformity in MRI data. IEEE transactions on medical imaging.

[49] MacDonald D, Kabani N, Avis D, Evans AC (2000). Automated 3-D extraction of inner and outer surfaces of cerebral cortex from MRI. NeuroImage.

[50] Kim JS (2005). Automated 3-D extraction and evaluation of the inner and outer cortical surfaces using a Laplacian map and partial volume effect classification. NeuroImage.

[51] Lerch JP, Evans AC (2005). Cortical thickness analysis examined through power analysis and a population simulation. NeuroImage.

[52] Robbins S, Evans AC, Collins DL, Whitesides S (2004). Tuning and comparing spatial normalization methods. Med Image Anal.

[53] Lyttelton O, Boucher M, Robbins S, Evans A (2007). An unbiased iterative group registration template for cortical surface analysis. NeuroImage.

[54] Kabani N, Le Goualher G, MacDonald D, Evans AC (2001). Measurement of cortical thickness using an automated 3-D algorithm: A validation study. Neuroimage.

[55] Lerch JP (2005). Focal decline of cortical thickness in Alzheimer’s disease identified by computational neuroanatomy. Cereb Cortex.

[56] Lee JK (2006). A novel quantitative cross-validation of different cortical surface reconstruction algorithms using MRI phantom. Neuroimage.

[57] Singh V (2006). Spatial patterns of cortical thinning in mild cognitive impairment and Alzheimer’s disease. Brain.

[58] La Joie R (2012). Region-specific hierarchy between atrophy, hypometabolism, and beta-amyloid (Abeta) load in Alzheimer’s disease dementia. J Neurosci.

[59] Jang H (2017). Correlations between Gray Matter and White Matter Degeneration in Pure Alzheimer’s Disease, Pure Subcortical Vascular Dementia, and Mixed Dementia. Sci Rep.

