# Author Correction: Multimodal imaging analyses in patients with genetic and sporadic forms of small vessel disease

**DOI:** 10.1038/s41598-019-51400-9

**Published:** 2019-10-15

**Authors:** Ko Woon Kim, Hunki Kwon, Young-Eun Kim, Cindy W. Yoon, Yeo Jin Kim, Yong Bum Kim, Jong Min Lee, Won Tae Yoon, Hee Jin Kim, Jin San Lee, Young Kyoung Jang, Yeshin Kim, Hyemin Jang, Chang-Seok Ki, Young Chul Youn, Byoung-Soo Shin, Oh Young Bang, Gyeong-Moon Kim, Chin-Sang Chung, Seung Joo Kim, Duk L. Na, Marco Duering, Hanna Cho, Sang Won Seo

**Affiliations:** 1 Department of Neurology, Samsung Medical Center, Sungkyunkwan University School of Medicine, Seoul, Korea; 2 Department of Neurology, Chonbuk National University Medical School & Hospital, Jeonju, Korea; 3 Department of Biomedical Engineering, Hanyang University, Seoul, Korea; 4 Department of Neurology, Yale University School of Medicine, New Haven, Connecticut USA; 5 Genome Research Center, Green Cross Genome, Yong-in, Korea; 6 Department of Neurology, Inha University School of Medicine, Incheon, Korea; 7 Department of Neurology, Chuncheon Sacred Heart Hospital, Hallym University College of Medicine, Chuncheon, Korea; 8 Department of Neurology, Kangbuk Samsung Hospital, Sungkyunkwan University School of Medicine, Seoul, Korea; 9 Department of Neurology, Kyung Hee University Hospital, Seoul, Korea; 10 Department of Neurology, Kangwon National University Hospital, Kangwon National University College of Medicine, Chuncheon, Korea; 11 Department of Laboratory Medicine and Genetics, Samsung Medical Center, Sungkyunkwan University School of Medicine, Seoul, Korea; 12 Department of Neurology, Chung-Ang University College of Medicine, Seoul, Korea; 13 Neuroscience Center, Samsung Medical Center, Seoul, Korea; 14 Department of Clinical Research Design & Evaluation, SAIHST, Sungkyunkwan University, Seoul, Korea; 15 Institute for Stroke and Dementia Research (ISD), University Hospital, LMU, Munich, Germany; 16 Department of Neurology, Gangnam Severance Hospital, Yonsei University College of Medicine, and Departments of, Clinical Research Design and Evaluation, Seoul, Korea

Correction to: *Scientific Reports* 10.1038/s41598-018-36580-0, published online 28 January 2019

The Acknowledgements section in this Article is incomplete.

“This research was supported by a National Research Foundation of Korea (NRF) grant funded by the Korean government (MSIP) (NRF-2017R1A2B2005081); and by the Brain Research Program through the National Research Foundation of Korea (NRF) funded by the Ministry of Science, ICT, & Future Planning (2016M3C7A1913844).”

should read:

“This research was supported by Research of Korea Centers for Disease Control and Prevention (2018-ER6203-01); the Brain Research Program through the National Research Foundation of Korea(NRF) funded by the Ministry of Science, ICT & Future Planning (2016M3C7A1913844); the National Research Foundation of Korea(NRF) grant funded by the Korea government(MSIP) (NRF-2017R1A2B2005081); and a grant from the Korean Health Technology R&D Project, Ministry of Health & Welfare, Republic of Korea(HI18C1629).”

