# Supplementary material for: Multimodal imaging analyses in patients with genetic and sporadic forms of small vessel disease
Source: Sci Rep. 2019 Jan 28;9:787. doi: 10.1038/s41598-018-36580-0 (PMC6349863; doi:10.1038/s41598-018-36580-0)
Supplement: Supplementary file 1 — Supplementary Figures [file 41598_2018_36580_MOESM1_ESM.pdf]

Multimodal imaging analyses in patients with genetic and sporadic forms of small vessel disease

Ko Woon Kim, MD, PhD,[1,2] Hunki Kwon, PhD,[3,4] Young-Eun Kim, MD, PhD,[5] Cindy W Yoon, MD, PhD,[6] Yeo Jin Kim, MD,[7] Yong Bum Kim, MD, PhD,[8] Jong Min Lee, PhD,[3] Won Tae Yoon, MD, PhD,[8] Hee Jin Kim, MD, PhD,[1] Jin San Lee, MD,[9] Young Kyoung Jang, MD,[1] Yeshin Kim, MD,[10] Hyemin Jang, MD,[1] Chang-Seok Ki, MD, PhD,[11] Young Chul Youn, MD, PhD,[12] Byoung-Soo Shin, MD, PhD,[2] Oh Young Bang, MD, PhD,[1] Gyeong-Moon Kim, MD, PhD,[1] Chin-Sang Chung, MD, PhD,[1] Seung Joo Kim, MD,[1] Duk L. Na, MD, PhD,[1,13,14] Marco Duering, MD, PhD,[15] Hanna Cho, MD, PhD[16*] and Sang Won Seo, MD, PhD[1,13,14] *

[1]Department of Neurology, Samsung Medical Center, Sungkyunkwan University School of Medicine, Seoul, Korea.
[2]Department of Neurology, Chonbuk National University Medical School & Hospital, Jeonju, Korea
[3]Department of Biomedical Engineering, Hanyang University, Seoul, Korea.
[4]Department of Neurology, Yale University School of Medicine, New Haven, Connecticut
[5]Genome Research Center, Green Cross Genome, Yong-in, Korea
[6]Department of Neurology, Inha University School of Medicine, Incheon, Korea
[7]Department of Neurology, Chuncheon Sacred Heart Hospital, Hallym University College of Medicine, Chuncheon, Korea
[8]Department of Neurology, Kangbuk Samsung Hospital, Sungkyunkwan University School of Medicine, Seoul, Korea
[9]Department of Neurology, Kyung Hee University Hospital, Seoul, Korea
[10]Department of Neurology, Kangwon National University Hospital, Kangwon National University College of Medicine, Chuncheon, Korea
[11]Department of Laboratory Medicine and Genetics, Samsung Medical Center, Sungkyunkwan University School of Medicine, Seoul, Korea
[12]Department of Neurology, Chung-Ang University College of Medicine, Seoul, Korea
[13]Neuroscience Center, Samsung Medical Center, Seoul, Korea
[14]Department of Clinical Research Design & Evaluation, SAIHST, Sungkyunkwan University, Seoul, Korea
[15]Institute for Stroke and Dementia Research (ISD), University Hospital, LMU, Munich, Germany
[16]Department of Neurology, Gangnam Severance Hospital, Yonsei University College of Medicine, and Departments of, Clinical Research Design and Evaluation, Seoul, Korea
***Corresponding author**

Sang Won Seo, MD, PhD

Department of Neurology, Samsung Medical Center, Sungkyunkwan University School of Medicine, 81 Irwon-ro, Gangnam-gu, Seoul, 06351, Korea.

Tel.: +82-2-3410-1233/-3599, Fax: +82-2-3410-0052, E-mail: sangwonseo@empal.com
Hanna Cho, MD, PhD

Department of Neurology, Gangnam Severance Hospital, Yonsei University College of Medicine, 211 Eonju-ro, Gangnam-gu, Seoul, 06273, Korea

Tel: +82-2-2019-3327, Fax: +82-2-3462-5904, E-mail: iguhanna@naver.com

Supplementary Figures

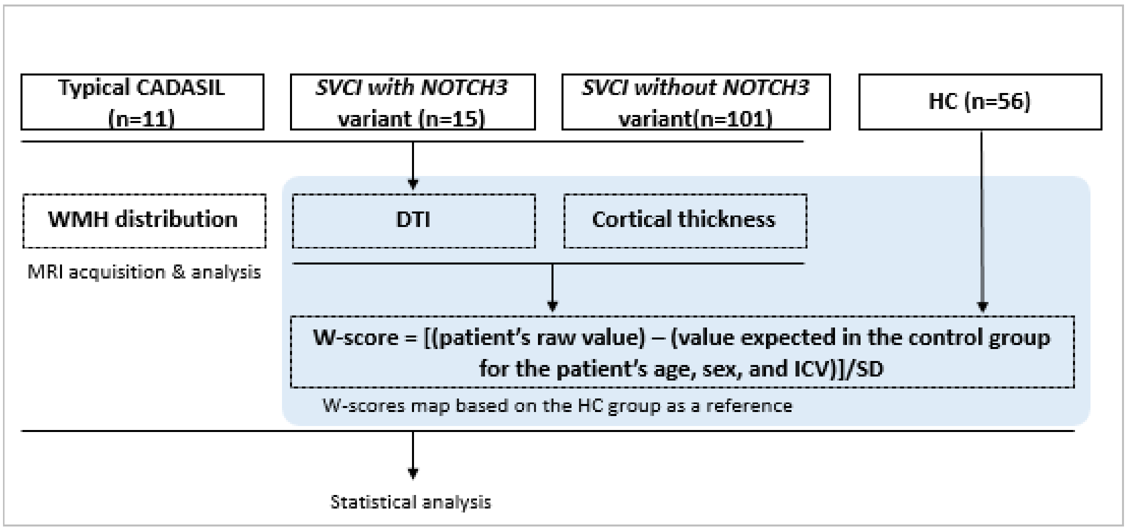

Supplementary Figure 1. Schematic diagram of the research process.

Typical CADASIL patients and SVCI patients (with and without *NOTCH3* variants) were recruited consecutively. Multimodal analysis was performed, consisting of WMH frequency mapping, DTI (TBSS), and cortical thickness measurements. DTI and cortical thickness data were transformed into W-scores corrected for age, sex, and ICV; the transformed scores were then analyzed statistically.

CADASIL: cerebral autosomal dominant arteriopathy with subcortical infarcts and leukoencephalopathy; DTI: diffusion tensor imaging; HC: healthy control; ICV: intracranial volume; SD: standard deviation; SVCI: subcortical vascular cognitive impairment; WMH: white matter hyperintensities

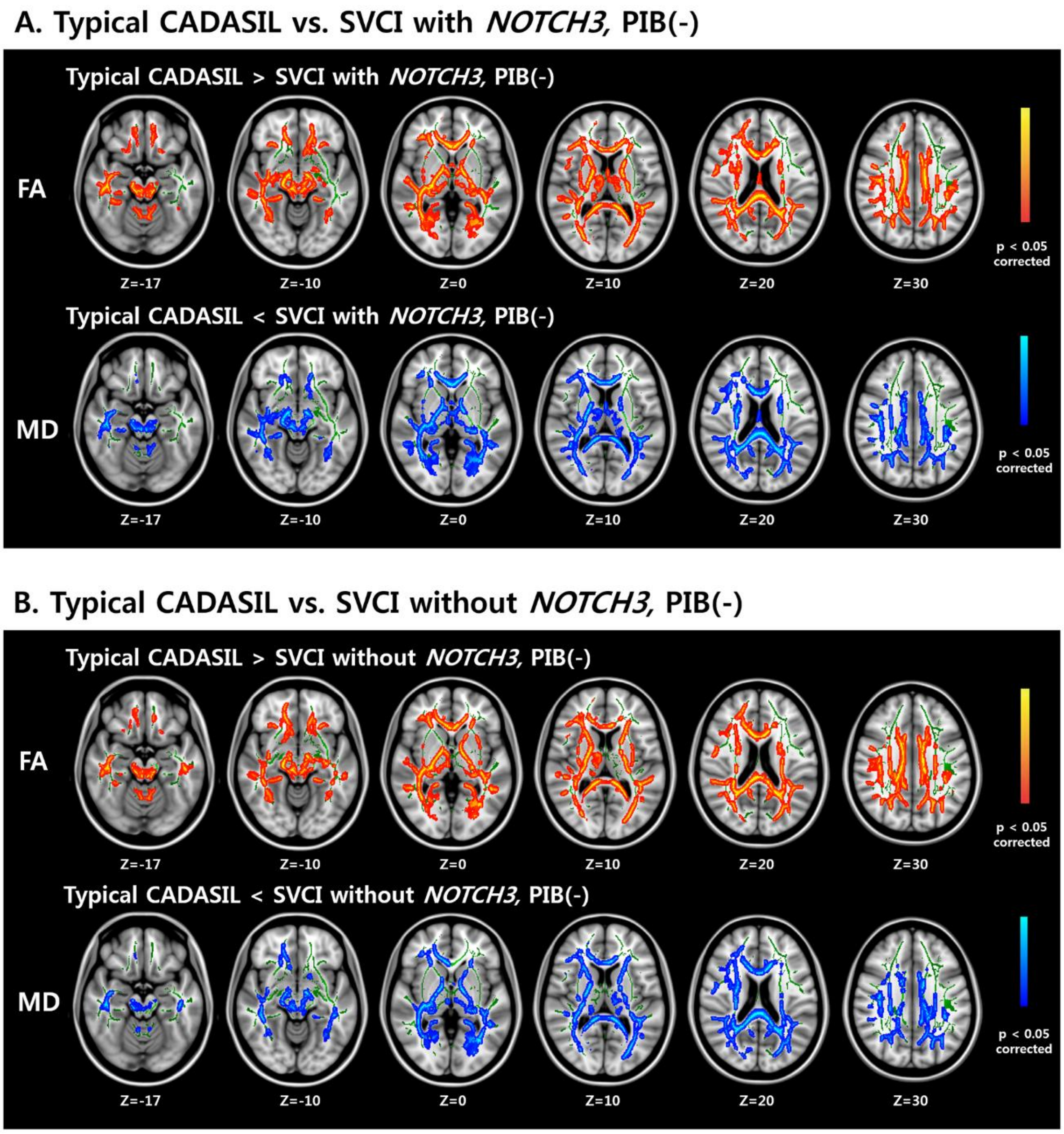

Supplementary Figure 2. Comparison of FA and MD W-scores between typical CADASIL group and PiB (-) SVCI groups.

SVCI patients (A) with *NOTCH3* variants and (B) without *NOTCH3* variants showing significantly lower FA (red-yellow color) and higher MD (Dark to light blue color) involved diffuse white matter tracts compared to typical CADASIL patients (W-score, $p < 0.05$, FWE-corrected).

CADASIL: cerebral autosomal dominant arteriopathy with subcortical infarcts and leukoencephalopathy; FA: fractional anisotropy; MD: mean diffusivity; PiB (-): negative Pittsburgh compound B PET; SVCI: subcortical vascular cognitive impairment.

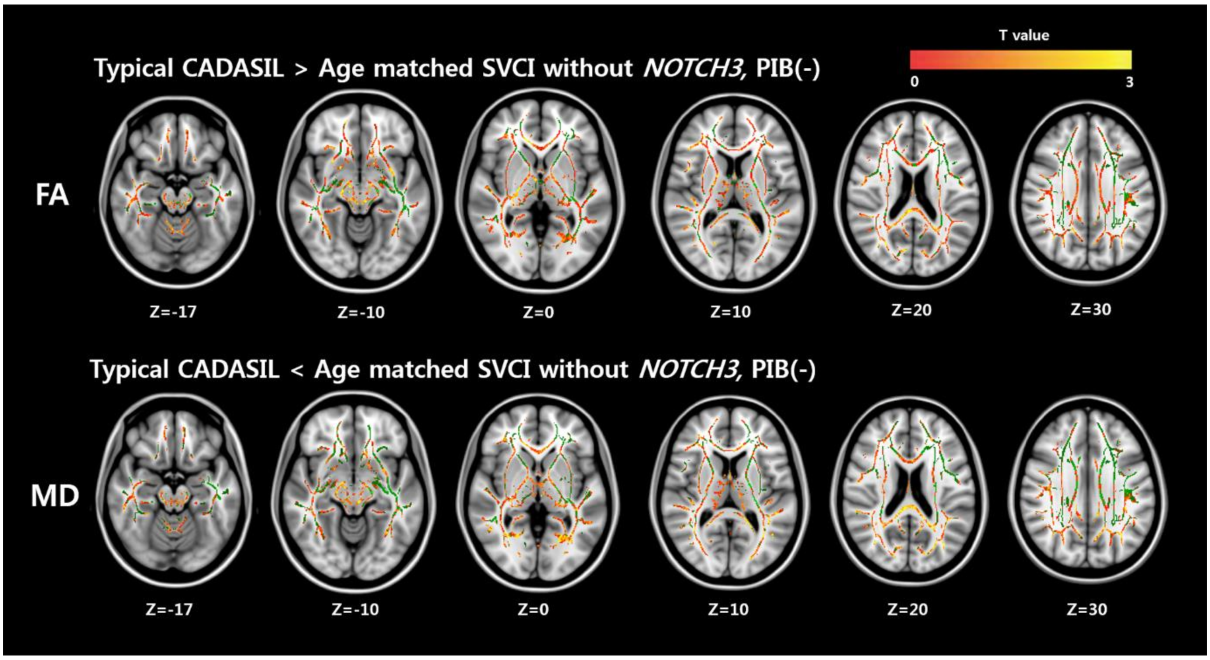

Supplementary Figure 3. Comparison of the FA and MD W-scores between age-matched typical CADASIL group and PiB (-) SVCI group without *NOTCH3* (W-score)

CADASIL: cerebral autosomal dominant arteriopathy with subcortical infarcts and leukoencephalopathy; FA: fractional anisotropy; MD: mean diffusivity; PiB (-): negative Pittsburgh compound B PET; SVCI: subcortical vascular cognitive impairment.

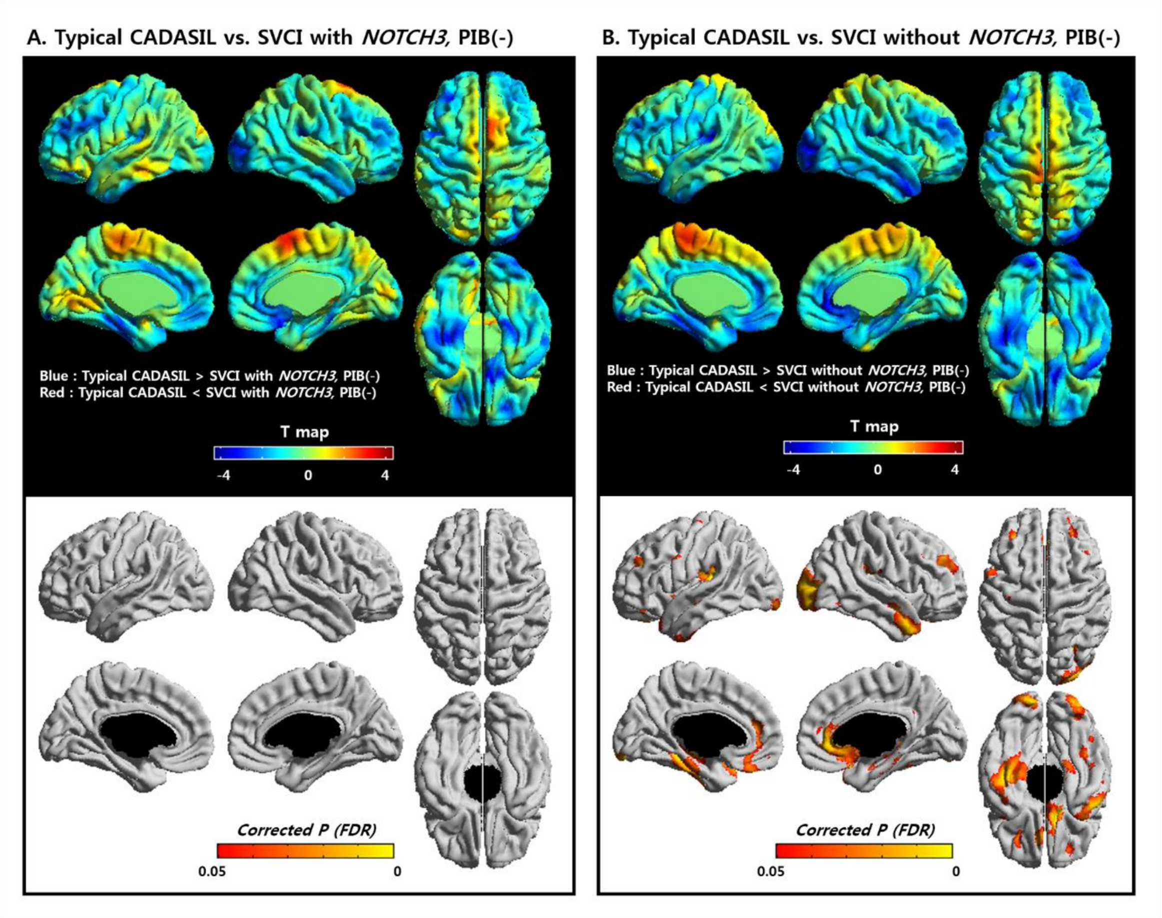

Supplementary Figure 4. Comparison of the cortical thickness pattern W-scores between typical CADASIL group and PiB (-) SVCI groups.

CADASIL: cerebral autosomal dominant arteriopathy with subcortical infarcts and leukoencephalopathy; PiB (-): negative Pittsburgh compound B PET; SVCI: subcortical vascular cognitive impairment.

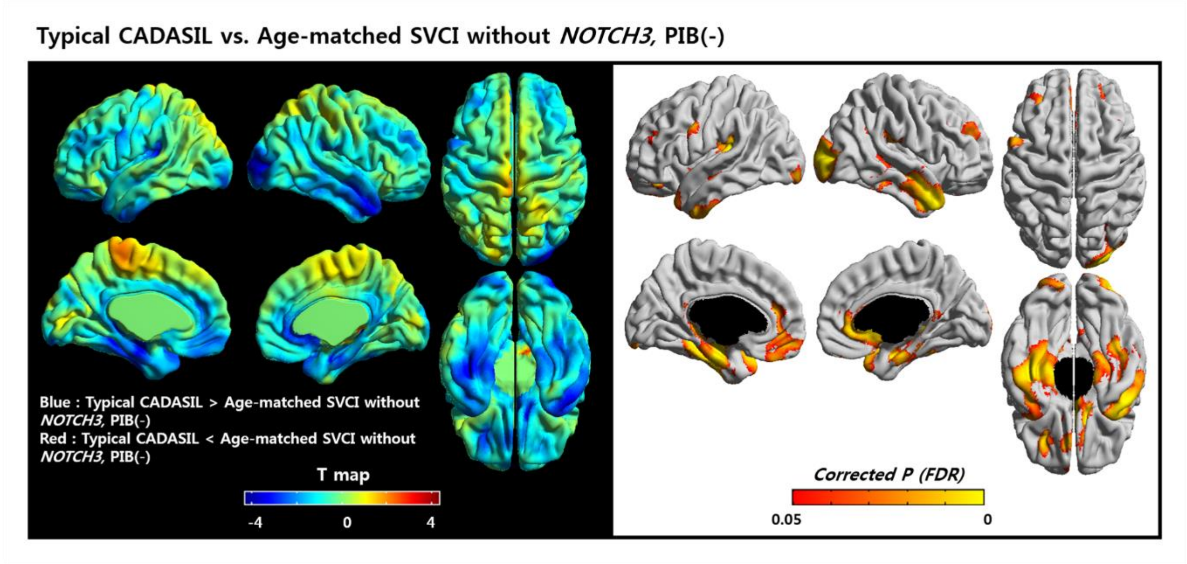

Supplementary Figure 5. Comparison of the cortical thickness pattern W-scores between age-matched typical CADASIL group and PiB (-) SVCI without *NOTCH3* variants group.

CADASIL: cerebral autosomal dominant arteriopathy with subcortical infarcts and leukoencephalopathy; PiB (-): negative Pittsburgh compound B PET; SVCI: subcortical vascular cognitive impairment.

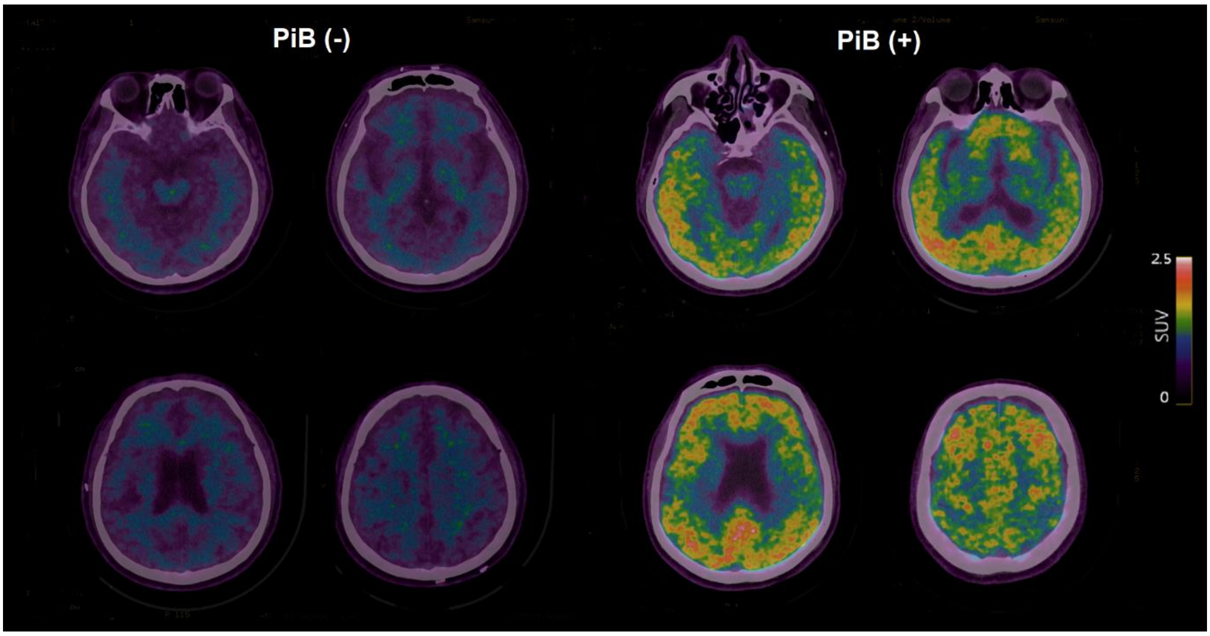

Supplementary Figure 6. PIB (-) and PIB (+) patients

PiB (-): negative Pittsburgh compound B PET; PiB (+): positive Pittsburgh compound B PET;
